# The first report of single nucleotide polymorphisms in the open reading frame of the prion-like protein gene in rabbits

**DOI:** 10.3389/fvets.2024.1388339

**Published:** 2024-06-17

**Authors:** Min-Ju Jeong, Yong-Chan Kim, Byung-Hoon Jeong

**Affiliations:** ^1^Korea Zoonosis Research Institute, Jeonbuk National University, Iksan, Jeonbuk, Republic of Korea; ^2^Department of Bioactive Material Sciences, Jeonbuk National University, Jeonju, Jeonbuk, Republic of Korea; ^3^Department of Biological Sciences, Andong National University, Andong, Republic of Korea

**Keywords:** prion, rabbit, Doppel, prion-like protein gene, *PRND*, polymorphism, SNP

## Abstract

**Background:**

Natural cases of prion disease have not been reported in rabbits, and prior attempts to identify a prion conversion agent have been unsuccessful. However, recent applications of prion seed amplifying experimental techniques have sparked renewed interest in the potential susceptibility of rabbits to prion disease infections. Among several factors related to prion disease, polymorphisms within the prion-like protein gene (*PRND*), a member of the prion protein family, have been reported as significantly associated with disease susceptibility in various species. Therefore, our study aimed to investigate polymorphisms in the *PRND* gene of rabbits and analyze their genetic characteristics.

**Methods:**

Genomic DNA was extracted from 207 rabbit samples to investigate leporine *PRND* polymorphisms. Subsequently, amplicon sequencing targeting the coding region of the leporine *PRND* gene was conducted. Additionally, linkage disequilibrium (LD) analysis was employed to assess the connection within and between loci. The impact of non-synonymous single nucleotide polymorphisms (SNPs) on the Doppel protein was evaluated using PolyPhen-2.

**Results:**

We found nine novel SNPs in the leporine *PRND* gene: c.18A > G, c.76G > C, c.128C > T, c.146C > T, c.315A > G, c.488G > A, c.525G > C, c.544G > A, and c.579A > G. Notably, seven of these *PRND* SNPs, excluding c.525G > C and c.579A > G, exhibited strong LD values exceeding 0.3. In addition, LD analysis confirmed a robust link between *PRNP* SNP c.234C > T and *PRND* SNPs at c.525G > C and c.579A > G. Furthermore, according to PolyPhen-2 and SIFT analyses, the four non-synonymous SNPs were predicted to have deleterious effects on the function or structure of the Doppel protein. However, PANTHER and Missense3D did not indicate such effects.

**Conclusion:**

In this paper, we have identified novel SNPs in the rabbit *PRND* gene and predicted their potential detrimental effects on protein function or structure through four non-synonymous SNPs. Additionally, we observed a genetic linkage between SNPs in the *PRND* and *PRNP* genes. These findings may provide insights into understanding the characteristics of rabbits as partially resistant species. To the best of our knowledge, this study is the first to genetically characterize *PRND* SNPs in rabbits.

## Introduction

Prion diseases are lethal neurodegenerative disorders characterized by aberrant misfolding of the cellular form of prion protein (PrP^C^) into the pathogenic, protease-resistant isoform of prion protein (PrP^Sc^), resulting in their accumulation within the brain (1, 2). Host species exhibiting high susceptibility to prion diseases include humans (Creutzfeldt-Jakob disease, CJD), deer and elk (chronic wasting disease, CWD), cattle (bovine spongiform encephalopathy, BSE), and sheep and goats (scrapie), whereas dogs, horses, and chickens are resistant to prion diseases (3, 4). Occurrences of prion disease have not been reported in these resistant species in natural environments, and experimental infections have also failed to induce the disease (4, 5).

To date, there have been no reported cases of natural prion disease infection in rabbits (4, 6). Despite experimental attempts involving infection with the human kuru and CJD agents, as well as the scrapie agent isolated from sheep and mouse (Me7 strain), rabbits have proven to be resistant (7, 8). Further supporting this resistance, *in vitro* experiments demonstrated that rabbit PrP^C^ did not convert to rabbit PrP^Sc^ in mouse neuroblastoma cells persistently infected with the mouse scrapie agent (RML strain) (9). Notably, a recent application of serial automated protein misfolding cyclic amplification (saPMCA) aimed to confirm protein misfolding and potential infection in rabbits, challenging the previous notion of rabbit resistance to prion diseases (10). The report indicated that rabbit brain homogenates, when amplified *in vitro* by saPMCA, exhibited resistance to proteinase K digestion. Upon injection into other rabbits, clinical symptoms emerged, leading to fatality. In brief, rabbits show prion resistance in natural transmission condition without extreme artificial replication techniques. Interestingly, rabbits exhibited a reduced propensity to transition to the β-structured state, which is believed to be associated with the mechanism of prion diseases, compared to susceptible species. However, they showed a higher propensity than dogs and horses (11). To further understand this partial resistance trait in rabbits, our investigation will focus on genetic factors associated with susceptibility to prion diseases.

Recent research has reported an association between the susceptibility to prion diseases and polymorphisms within the prion gene family (12–22). The prion protein gene family comprises the prion protein gene (*PRNP*), prion-like protein gene (*PRND*), prion-related protein gene (*PRNT*), and the shadow of prion protein gene (*SPRN*) (23). Among them, special attention is being given to *PRND* as a notable candidate gene. The *PRND* gene is located most closely to the *PRNP* gene, and the Doppel protein, encoded by *PRND*, shares biochemical and structural similarities with PrP (24, 25).

Previous studies propose an association between *PRND* polymorphisms and prion disease susceptibility in several species (26–30). In humans, significant differences in the frequencies of polymorphisms at codon 174 and the 3′ untranslated region (UTR) +28 of human *PRND* were observed in sporadic CJD patients compared to healthy controls (26, 27). In cattle, the genotype distributions of polymorphisms at codons 95 and 132 of the bovine *PRND* gene were significantly different between BSE-affected and healthy German Fleckvieh cattle (28). In sheep, linkage disequilibrium (LD) analysis revealed a significant linkage between the G allele of codon 26 of the ovine *PRND* gene and the ARR allele of the ovine *PRNP* gene, known to confer genetic resistance to scrapie (29). Even in goats, caprine *PRND* single nucleotide polymorphisms (SNPs) c.28 T *>* C, c.151A *>* G, and c.385G *>* C are strongly linked to caprine *PRNP* c.428A > G (H143R) (30). Consequently, the major homozygote genotype of caprine *PRND* SNPs is genetically associated with the caprine *PRNP* HH genotype, which is related to scrapie progression.

The exploration of polymorphisms in the *PRND* gene of prion disease-resistant species has yielded intriguing findings. In the canine *PRND* gene, four polymorphisms were identified, and researchers attempted to validate the association between *PRNP* and *PRND* through LD analysis (31). Despite the relatively shorter genetic distances in dogs compared to sheep and goats, no strong LD was observed between *PRNP* and *PRND*. In the equine *PRND* gene, SNPs were either absent or rare, depending on the breed (32, 33). Even in cases where they were rarely present, weak LD was confirmed between *PRNP* and *PRND* (33). Moreover, the *PRND* gene has not been identified in birds (34).

To date, studies on *PRND* polymorphisms in rabbits have not yet been performed, and this area is considered worth exploring. In this study, we investigated the genotype and allele frequencies of *PRND* polymorphisms in a group of 207 rabbits. In addition, we performed an LD analysis between *PRNP* and *PRND* to identify genetic linkage. Furthermore, we assessed the possible impact of non-synonymous SNPs on the structure and function of the Doppel protein using *in silico* prediction tools.

## Materials and methods

### Sample preparation

All 207 rabbit samples of hybrid breeding rabbits (New Zealand white and Flemish Giant FG) were provided by a slaughterhouse located in the Republic of Korea. Genomic DNA was isolated from 20 mg brain tissue following the manufacturer’s manuals using the Labopass Tissue Genomic DNA Isolation Kit (Cosmo Genetech Co., Ltd., Seoul). The overall experimental processes were approved by the Jeonbuk National University Institutional Animal Care and Use Committee (CBNU 2019-058). All experiments were performed in accordance with the Korea Experimental Animal Protection Act.

### Genetic analysis in the leporine *PRND* gene

Primers were designed based on the leporine *PRND* gene sequence (*Oryctolagus cuniculus*) available in GenBank at the National Center for Biotechnology Information (NCBI) (Gene ID: 100347890). The gene-specific forward and reverse primer sequences were GGGTAGACCGGTTGGGAAAT and TGAGCACTGAAGCACTGAGG, respectively. Polymerase chain reaction (PCR) was performed targeting the coding region of the leporine *PRND* gene by an S-1000 Thermal Cycler (Bio-Rad, Hercules, CA, United States). The PCR conditions followed the manual guide of BioFACT™ Taq DNA Polymerase (BioFACT Co., Ltd., Daejeon, Korea) with an annealing temperature of 65°C. The amplified products were purified using the FavorPrep™ GEL/PCR Purification Kit (Favorgen Biotech Corp., Kaohsiung, Taiwan), and then sequenced by an ABI PRISM 3730XL Analyzer (ABI, Foster City, CA, United States). These sequencing results for each sample were analyzed using Finch TV software (Geospiza Inc., Seattle, WA, United States).

### Statistical analysis

The Hardy–Weinberg Equilibrium (HWE) test was conducted to assess the genotyping errors of the collected individual samples for this study. In HWE testing, a *p*-value lower than 0.05 indicates that the observed genotype or allele frequencies are not consistent with HWE (35, 36). The HWE test was conducted by the Michael H. Court’s calculator.

Additionally, LD analysis was performed to examine the statistical relationship between SNPs at each locus of the genes. LD, a measure of the correlation between two genetic loci (or SNPs), was assessed using the *r*^2^ value (37). The *r*^2^ value ranges from 0 to 1. Higher values indicate strong linkage between two genetic loci, suggesting the presence of associated genetic regions. Conversely, lower *r*^2^ values indicate that the variations at two genetic loci are independent or weakly correlated. The LD and haplotype distribution were estimated using the Haploview version 4.2 (Broad Institute, Cambridge, MA, United States).

### *In silico* prediction of the impact of non-synonymous SNPs in leporine *PRND*

PolyPhen-2 determines the effect of non-synonymous SNPs on the structure or function of a protein according to a position-specific independent counts (PSIC) score difference. The results are assigned as “probably damaging,” “possibly damaging” or “benign,” depending on the degree of risk. SIFT predicts the impact of amino acid substitutions on protein function based on sequence homology, assuming that alignment correlates well with evolution to maintain protein function. The SIFT score ranges from 0 to 1, where values below 0.05 are considered deleterious. PANTHER assesses the effect of non-synonymous SNPs on function using PANTHER-PSEP (position-specific evolutionary preservation). Positions conserved over longer periods are expected to have more detrimental effects. These effects are quantitatively scored as Pdel (probability of deleterious effect), and the results are classified as “probably damaging,” “possibly damaging,” and “probably benign.” Missense3D predicts structural changes resulting from deleterious variants that affect protein stability. It identifies structural damage through comprehensive analysis, which includes examining factors such as disruption of buried salt bridges and alterations in secondary structure.

### Analysis of the genetic linkage among SNPs of *PRNP* and *PRND* genes

To investigate the genetic linkage between *PRNP* and *PRND* SNPs, we conducted LD analysis between the loci of these two genes. Initially, we obtained the dataset of *PRNP* genotypes from rabbits, which included results from previously reported 203 samples. Subsequently, we performed preprocessing to match the *PRNP* genotype data with the individuals analyzed for *PRND*. Of the total genotype datasets examined, 201 matched, and these were used to analyze the LD scores between *PRNP* and *PRND* SNPs.

## Results

### Investigation of leporine *PRND* polymorphisms

**Figure 1:**
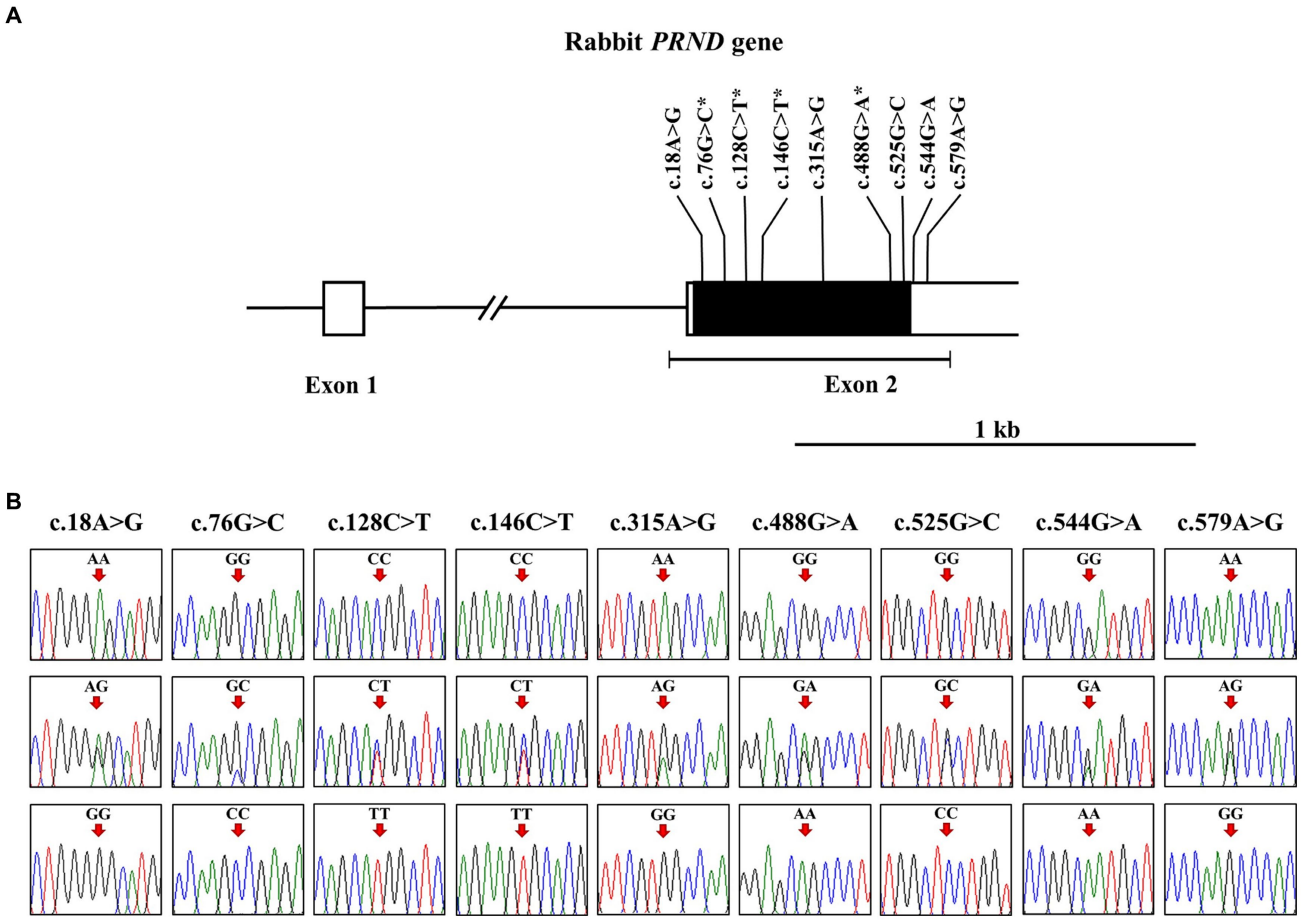
Identification of single nucleotide polymorphisms (SNPs) in the leporine prion-like protein gene (*PRND*). **(A)** The schematic diagram illustrates the genomic structure of the leporine *PRND*. The open reading frame (ORF) within exon 2 is represented by the black box, and the 5′ and 3′ untranslated regions (UTRs) from exon 1 to 2 are shown by the white boxes. The edged horizontal bar indicates the length of PCR products in this study. The positions of the polymorphisms identified in this study are shown in bold, with an asterisk denoting the non-synonymous SNP. **(B)** Electropherograms displaying nine novel SNPs discovered in the leporine *PRND* gene are presented. The electropherograms show three genotypes at c.18A > G, c.76G > C, c.128C > T, c.146C > T, c.315A > G, c.488G > A, c.525G > C, c.544G > A, and c.579A > G. The colors of the peaks represent each base of the DNA sequence as follows: green for adenine; red for thymine; blue for cytosine; black for guanine. Arrows indicate the position of the polymorphisms identified in this study. Upper panel, homozygote of the major allele; middle panel, heterozygote; lower panel, homozygote of the minor allele.

To investigate the leporine *PRND* polymorphisms, we analyzed DNA sequences targeting the open reading frame (ORF) of leporine *PRND* in 207 rabbits. The leporine *PRND* gene comprises two exons, with the ORF (537 bp) located in exon 2 (Figure 1A). PCR and sequencing were performed using a pair of primers designed in this study, and the sequencing results were identical to the leporine *PRND* gene registered in GenBank (Gene ID: 100347890). We found nine novel SNPs in leporine *PRND*: c.18A > G, c.76G > C, c.128C > T, c.146C > T, c.315A > G, c.488G > A, c.525G > C, c.544G > A, and c.579A > G (Figure 1B). Among them, four SNPs at c.76G > C (A26P), c.128C > T (T43M), c.146C > T (A49V), and c.488G > A (R163Q) are nonsynonymous SNPs within the ORF region, and two SNPs at c.544G > A and c.579A > G are located in the 3′ untranslated region (Figure 1A). Detailed information about the genotype and allele frequencies of the leporine *PRND* SNPs is described in Table 1.

**Table 1:** Genotype and allele frequencies of the leporine prion-like protein gene (*PRND*) single nucleotide polymorphisms (SNPs).

Polymorphism	Genotype frequency, *n* (%)			Total, *n* (%)	Allele frequency, *n* (%)		Total, *n* (%)	HWE
c.18A > G	**AA**	**AG**	**GG**		**A**	**G**		
(G6G)	57	106	44	207	220	194	414	0.68
	(27.54)	(51.21)	(21.26)	(100)	(53.14)	(46.86)	(100)	
c.76G > C	**GG**	**GC**	**CC**		**G**	**C**		
(A26P)	57	106	44	207	220	194	414	0.68
	(27.54)	(51.21)	(21.26)	(100)	(53.14)	(46.86)	(100)	
c.128C > T	**CC**	**CT**	**TT**		**C**	**T**		
(T43M)	55	110	42	207	220	194	414	0.33
	(26.57)	(53.14)	(20.29)	(100)	(53.14)	(46.86)	(100)	
c.146C > T	**CC**	**CT**	**TT**		**C**	**T**		
(A49V)	55	110	42	207	220	194	414	0.33
	(26.57)	(53.14)	(20.29)	(100)	(53.14)	(46.86)	(100)	
c.315A > G	**AA**	**AG**	**GG**		**A**	**G**		
(V105V)	55	109	43	207	219	195	414	0.41
	(26.57)	(52.66)	(20.77)	(100)	(52.90)	(47.10)	(100)	
c.488G > A	**GG**	**GA**	**AA**		**G**	**A**		
(R163Q)	55	110	42	207	220	194	414	0.33
	(26.57)	(53.14)	(20.29)	(100)	(53.14)	(46.86)	(100)	
c.525G > C	**GG**	**GC**	**CC**		**G**	**C**		
(L175L)	183	23	1	207	389	25	414	0.76
	(88.41)	(11.11)	(0.48)	(100)	(93.96)	(6.04)	(100)	
c.544G > A	**GG**	**GA**	**AA**		**G**	**A**		
	56	108	43	207	220	194	414	0.49
	(27.05)	(52.17)	(20.77)	(100)	(53.14)	(46.86)	(100)	
c.579A > G	**AA**	**AG**	**GG**		**A**	**G**		
	183	23	1	207	389	25	414	0.76
	(88.41)	(11.11)	(0.48)	(100)	(93.96)	(6.04)	(100)	

We investigated the extent of LD among the leporine *PRND* SNPs by calculating the *r*^2^ values (Table 2). Seven of the *PRND* SNPs, excluding c.525G > C and c.579A > G, exhibited strong linkage with values greater than 0.3. In addition, we examined the haplotype frequency of the leporine *PRND* SNPs (Table 3). The most frequently observed haplotype was GCCCAGGGA (46.9%), followed by AGTTGAGAA (46.6%), AGCCAGCGG (6%), AGTTGAGGA (0.2%), and AGCCGGGAA (0.2%).

**Table 2:** Linkage disequilibrium (LD) scores of the leporine prion-like protein gene (*PRND*) single nucleotide polymorphisms (SNPs).

*r*^2^	c.18A > G	c.76G > C	c.128C > T	c.146C > T	c.315A > G	c.488G > A	c.525G > C	c.544G > A	c.579A > G
c.18A > G	–	**1.0**	**0.778**	**0.778**	**0.758**	**0.778**	0.057	**0.778**	0.057
c.76G > C	–	–	**0.778**	**0.778**	**0.758**	**0.778**	0.057	**0.778**	0.057
c.128C > T	–	–	–	**1.0**	**0.99**	**1.0**	0.057	**0.981**	0.057
c.146C > T	–	–	–	–	**0.99**	**1.0**	0.057	**0.981**	0.057
c.315A > G	–	–	–	–	–	**0.99**	0.057	**0.99**	0.057
c.488G > A	–	–	–	–	–	–	0.057	**0.981**	0.057
c.525G > C	–	–	–	–	–	–	–	0.057	**0.1**
c.544G > A	–	–	–	–	–	–	–	–	0.057
c.579A > G	–	–	–	–	–	–	–	–	–

Bold indicates strong LD with *r*^2^ > 0.3.

**Table 3:** Haplotype frequencies of the leporine prion-like protein gene (*PRND*) single nucleotide polymorphisms (SNPs).

Haplotype	Frequency, *n* (%)
GCCCAGGGA	194 (46.9)
AGTTGAGAA	192 (46.6)
AGCCAGCGG	24 (6)
AGTTGAGGA	2 (0.2)
AGCCGGGAA	2 (0.2)

### *In silico* prediction of the functional effect of non-synonymous leporine *PRND* SNPs

We evaluated the potential effect of non-synonymous SNPs on leporine *PRND* using *in silico* prediction tools (Table 4). Both PolyPhen-2 and SIFT predicted that all four non-synonymous SNPs would have deleterious effects on protein function and structure. In Polyphen-2, the three SNPs at c.76G > C (A26P), c.128C > T (T43M), and c.488G > A (R163Q) were predicted as “Possibly damaging” with scores of 0.895, 0.924, and 0.816, respectively. Interestingly, the SNP at c.146C > T (A49V) was predicted as “Probably damaging” with a score of 0.991. In SIFT, all non-synonymous SNPs were predicted to impact protein function with a score of 0.00. However, both PANTHER and Missense3D classified all non-synonymous SNPs as benign.

**Table 4:** *In silico* prediction of the functional effect of non-synonymous single nucleotide polymorphisms (SNPs) in the leporine prion-like protein gene (*PRND*).

Variation	PolyPhen-2		SIFT		PANTHER		Missense3D
	Score	Prediction	Score	Prediction	Score	Prediction	Prediction
c.76G > C (A26P)	0.895	Possibly damaging	0	Damaging	–	Not scored	No structural damage detected
c.128C > T (T43M)	0.924	Possibly damaging	0	Damaging	0.19	Probably benign	No structural damage detected
c.146C > T (A49V)	0.991	Probably damaging	0	Damaging	0.27	Probably benign	No structural damage detected
c.488G > A (R163Q)	0.816	Possibly damaging	0	Damaging	0.27	Probably benign	No structural damage detected

### Investigation of genetic linkage between leporine *PRNP* and *PRND* SNPs

A previous study reported a synonymous SNP (c.234C > T) identified in the ORF of the leporine *PRNP* gene (38). To investigate whether leporine *PRND* SNPs have strong genetic linkage with this leporine *PRNP* SNP, we performed LD analysis between *PRNP* and *PRND* SNPs using *r*^2^ values. LD scores were estimated in 201 animals after excluding 6 animals that did not have genotyping data for the *PRNP* gene. As shown in Table 5, *PRND* SNPs at c.525G > C and c.579A > G were strongly linked with *PRNP* SNP c.234C > T (*r*^2^ value of 0.912). The remaining seven *PRND* SNPs were weakly linked with *r*^2^ scores of less than 0.3.

**Table 5:** Linkage disequilibrium (LD) scores between leporine prion protein gene (*PRNP*) and prion-like protein gene (*PRND*) single nucleotide polymorphisms (SNPs).

*r*^2^	*PRND*								
	c.18A > G	c.76G > C	c.128C > T	c.146C > T	c.315A > G	c.488G > A	c.525G > C	c.544G > A	c.579A > G
*PRNP* c.234C > T	0.052	0.052	0.05	0.05	0.051	0.05	**0.912**	0.05	**0.912**

Bold indicates strong LD with *r*^2^ > 0.3.

## Discussion

Since the pathological mechanisms of prion diseases remain elusive, investigating the genetic characteristics of prion disease-susceptible species is crucial for understanding this disease. However, exploring the genetic characteristic of resistant species offers a novel avenue to unravel the mysteries of prion diseases, providing valuable perspective (4). Previous research has suggested that resistance to prion diseases may be attributed to a specific structural stability unique to PrP in species resistant to such disease. For instance, the presence of an aspartic acid (Asp) residue at codon 163 of canine PrP enhances protein stability by forming an additional salt bridge compared to asparagine (Asn), the amino acid found at that position in prion disease-susceptible animal PrP (39, 40). This structural stabilization contributes to the resistance properties. Similarly, the Asp residue at codon 167 in equine PrP plays a crucial role in maintaining the well-defined structure of PrP, particularly within the β2–α2 loop, thereby contributing to disease resistance (41–44). While the amino acid structure of rabbit PrP shares similarities with that of prion disease-susceptible species, a detailed examination revealed that the β-sheet of rabbit PrP is shorter than that of human PrP (6). Notably, the β2–α2 loop of rabbit PrP spanning residues 165–175 exhibits a well-defined structure, contributing to the structural stability of rabbit PrP (11, 45). The V166 residue establishes hydrophobic contacts with the Y218 residue of helix 3 in rabbit PrP, potentially influencing the structural stability of the β2–α2 loop (11, 45). Furthermore, the S174 residue in rabbit PrP forms a robust hydrogen bond with the N171 residue, a key interaction believed to play a role in the observed resistance to prion susceptibility when serine replaces asparagine at codon 174 in mouse PrP (9, 11).

Given the influence of rabbit PrP-specific residues on structural stability, our attention may be directed towards exploring alterations in the rabbit PrP sequence and the potential influencing factors. In a recent study investigating polymorphisms within rabbit *PRNP*, a single SNP within the ORF region was identified. The study assessed the impact of substitutions in amino acids unique to rabbit compared to those conserved in other prion disease-susceptible species (38). While *in silico* analysis predicted benign effects on protein function and structure for amino acid substitutions in the unique residues of rabbit PrP (38), 3D structure predictions revealed weakened hydrogen bonds in residues such as S175, Q221, A226, and A230 of rabbit PrP resulting from these substitutions (38). However, the sensitivity to prion diseases cannot be fully explained by analyzing the sequence characteristics of *PRNP* alone. Recent studies have reported that the genetic profile of polymorphisms in the *PRND* gene, a member of the prion protein family, serves as an important cofactor associated with susceptibility to various types of prion diseases (12–22, 26–33, 46–53).

Recent case-controlled studies have suggested that susceptibility to prion diseases is associated with *PRND* polymorphisms at codon 174 and 3′ untranslated region (UTR) +28 in humans (12, 26, 27), codons 95 and 132 in cattle (28), codon 26 in sheep (29), and codon 10 in goats (54). Moreover, significant associations have been observed between polymorphisms of the *PRNP* and *PRND* genes in sheep and goats (29, 30). This suggests that *PRND* SNPs may indirectly contribute to susceptibility to prion diseases across various species. In our study, we investigated polymorphisms within the ORF region of the leporine *PRND* gene, which is located proximal to the *PRNP* gene and observed a strong linkage between *PRNP* (c.234C > T) and *PRND* (c.525G > C and c.579A > G) polymorphisms in rabbits. These results align with prion disease-susceptible species such as goats and sheep (strong LD between *PRNP* and *PRND* SNPs). Interestingly, weak LD values were observed for seven *PRND* SNPs (c.18A > G, c.76G > C, c.128C > T, c.146C > T, c.315A > G, c.488G > A, and c.544G > A). Particularly, weak linkage between *PRNP* and *PRND* SNPs was observed in dogs and horses, which can be interpreted as a characteristic of prion disease-resistant species. Although natural infections have not been reported in rabbits, they are considered partially resistant species with confirmed experimental infection potential. Therefore, further research is needed to explore various characteristics related to genetic diversity in rabbits. Additional research on the association of intergenic polymorphisms in rabbits, considering the observed weak LD in resistant species and strong LD in susceptible species, will require in depth exploration.

In addition, we identified several SNPs, and four non-synonymous SNPs showed a potent effect on leporine Doppel (Figure 1; Table 4). All non-synonymous SNPs were predicted to impact protein function by PolyPhen-2 and SIFT, whereas PANTHER and Missense3D did not indicate any effect. Since *in silico* prediction tools employ algorithms based on their specific criteria, further research is needed to validate these predictions using cellular or animal models in the future. Interestingly, since the Doppel protein is predominantly expressed in the testis, the impact induced by the *PRND* SNP may affect male fertility (55, 56). In the previous study, researchers investigated the influence of a single SNP, c.78G.A (A26A), identified in the *PRND* gene of sheep, on sperm reproductive ability (29, 57). Semen from sheep carrying the A allele exhibited a higher proportion of the F pattern in spermatozoa compared to those carrying the G allele. Additionally, there was an improvement in cleavage rate and enhanced embryo rates at 6 and 8 days. Consequently, the A allele at codon 26 of the *PRND* gene has been suggested to correlate with male reproductive performance. Although individuals carrying SNPs that may significantly impact reproductive capacity could have been culled from the breeding population, the degree of variation in reproductive ability may depend on the extent to which *PRND* SNPs affect the functional characteristics of Doppel protein. Therefore, further functional studies in natural rabbit populations, which lack the artificial selection present in the breeding population used in this study, are needed to elucidate the relationship between *PRND* polymorphism and reproductive capacity.

In this study, we investigated the polymorphism of the leporine *PRND* gene and analyzed its characteristics. The hybrid breeding rabbits we utilized are primarily consumed breeds in Korea. All individuals included in this study were sourced from rabbits slaughtered at regional abattoirs of the Korea Rabbit & Deer Farmers National Agricultural Cooperative Federation during sample collection. To determine whether rabbits from different breeds exhibit genotype distributions or novel polymorphisms distinct from those observed in this study, future genetic analysis studies on various rabbit breeds, particularly native breeds, will be necessary.

## Conclusion

In the present study, we found nine novel SNPs in the leporine *PRND* gene, including four deleterious non-synonymous SNPs. In addition, we performed LD analysis between *PRNP* and *PRND* polymorphisms and found strong LD between *PRNP* (c.234C > T) and *PRND* SNPs (c.525G > C and c.579A > G). The remaining seven *PRND* SNPs (c.18A > G, c.76G > C, c.128C > T, c.146C > T, c.315A > G, c.488G > A, and c.544G > A) showed weak linkage with *PRNP* SNP. To the best of our knowledge, this study is the first to present a genetic characterization of *PRND* SNPs in rabbits.

## Data availability statement

The data presented in the study are deposited in the DRYAD repository (https://datadryad.org/stash/share/3gw51vGYS1FWIFzOBEC5JEfIhPxygvyC9eAgSh3SwKI).

## Ethics statement

The animal study was approved by the overall experimental processes were approved by the Jeonbuk National University Institutional Animal Care and Use Committee (CBNU 2019-058). All experiments were performed in accordance with the Korea Experimental Animal Protection Act. The study was conducted in accordance with the local legislation and institutional requirements.

## Author contributions

M-JJ: Conceptualization, Formal analysis, Writing – original draft, Writing – review & editing. Y-CK: Conceptualization, Formal analysis, Writing – review & editing. B-HJ: Conceptualization, Formal analysis, Writing – review & editing.
